# *Psallops niedzwiedzkii*, a new species from Ghana with a key to African species (Heteroptera, Miridae, Psallopinae)

**DOI:** 10.3897/zookeys.603.6978

**Published:** 2016-07-07

**Authors:** Aleksander Herczek, Yuri A. Popov, Jacek Gorczyca

**Affiliations:** 1 Silesian University, Department of Zoology, 40-Katowice, Bankowa 9, Poland; 2 Borissiak Paleontological Institute, Russian Academy of Sciences, Profsoyuznaya Str. 123, 117997 Moscow

**Keywords:** Heteroptera, Miridae, Psallopinae, Psallops, new species, Africa

## Abstract

A new species from Ghana, *Psallops niedzwiedzkii* Herczek & Popov, **sp. n.** is described. The dorsal habitus, head and male genitalia are presented and some morphological features are discussed. A key, short descriptions and map of the distribution of the African species of the genus are also provided.

## Introduction

The small plant bug subfamily Psallopinae is most probably a relict group that is closely related to the subfamily Isometopinae. Schuh and Schwarz (1984) and Cassis and Schuh (2012) believed that Isometopinae, Psallopinae and Cylapinae should constitute a single clade. Currently, these subfamilies are considered to be the primitive groups among the other Miridae. The geographical distribution and life history of Psallopinae is still poorly known, although eight species from Asia were recently added (Lin 2004, Yasunaga 1999, Yasunaga et al. 2010). Bionomical data are known for 11 species in the subfamily: seven were caught using a light trap, three were found on plants, two were caught with a sweep net and two by using Malaise traps. Details about their habitats and habits are also poorly known. Usinger (1946) reported *Psallops oculatus* Usinger, 1946 on *Asplenium nidus* Linne, 1753 (Polypodiales: Aspleniaceae). Some psallopinous bugs from Thailand have been found under half-detached bark fragments of fabaceous broadleaf plants (Yasunaga et al. 2010). Only *Psallops myiocephalus* Yasunaga, 1999 from Japan is known from the oak *Quercus acutissima* Carruth, 1862 (Fagaceae) in the Nagasaki Prefecture of Kyushu (Yasunaga 1999). These insects probably have nocturnal habits. They all have a small body size, i.e. 1.73-3.5 mm. All of the species that have recently been described have been placed in the genus *Psallops* Usinger, 1946.

To date, only two species that belong to the subfamily Psallopinae have been described from Africa, *Psallops webbii* Herczek & Popov, 2014 (Herczek and Popov 2014) and *Psallops linnavuorii* Herczek, Popov & Gorczyca, 2016 (Herczek et al. 2016). *Psallops webbii* was collected by R. E. Linnavuori in Igboho-Kiohi (the northern part of Oyo province in western Nigeria) in July 1973 (the second specimen of *Psallops webbii* comes from Equatoria (south Sudan) and was collected by R.E. Linnavuori in April 1963). *Psallops linnavuorii* was collected by Leston in Ghana in November 1965 (Fig. 8).

## Material and methods

The species was encountered in the collection of the Museum in Copenhagen. *Psallops niedzwiedzkii* sp. n. was collected from a forest habitat in October 1965 by L. R. Cole. It belongs to D. Leston’s collection (coll. 1976-5093). The abdomen and aedeagus had already been dissected and placed in a separate vial under the specimen. When describing the species, the genitalic structures were transferred to KOH, chloroaldehyde and finally to chloralphenol. After the examination, the structures were immersed in a drop of Berlese liquid on a celluloid board and attached underneath the specimen. The original vial did not contain the right paramere. Colour photographs and drawings were obtained using a Nikon Eclipse E 600 microscope and the computer program NIS Elements, Ver. 4.10. Measurements were taken with a micrometer and are presented in millimetres (mm). The proportions of the selected body parts are presented in Table 1. The terminology used for the male genitalia follows Konstantinov (2003).

**Table 1.** Proportions of the selected male body parts of African species.

	*Psallops niedzwiedzkii*	*Psallops linnavuorii*	*Psallops webbii*
Body length / width	2.67	2.70	2.74
Head width / length	2.7	2.48	2.79
Eye dorsal width / vertex width	2.0	2.0	1.12
Head width / vertex width	4.5	5.18	2.79
Head width / pronotum width	0.68	0.76	0.62
Antennal segments II: I	3.78	4	-
II antennal segment length / pronotum width	0.67	0.91	-
Pronotum width / head width	1.46	1.32	1.62
Pronotum length / head length	1.6	1.35	1.58
Pronotum posterior / anterior length	2.26	1.53	2.0
Pronotum width / length	2.47	3.08	2.87
Pronotum length / commissurae claval length	0.76	0.72	0.83
Mesoscutum + scutellum length / pronotum length	1.03	1.19	1.37
Scutellum length / Mesoscutum length	3.12	3.44	3.1
Commissurae claval length / mesoscutum + scutellum length	1.27	1.16	0.88
Corium length / cuneus length	3.27	3.53	3.17
Hind femur length / width	3.73	3.04	3.0
Hind tibia length / femur length	1.12	1.62	1.47
Hind tibia length / pronotum width	1.16	1.64	1.33
Tibia length / tarsus length	2.71	3.97	4.60
Hind tarsus II: I	2.6	2.08	1.90
Cell length / width	2.22	2.59	2.39

## Taxonomy

### Genus *Psallops* Usinger, 1946: 86.

Type species by original designation *Psallops oculatus* Usinger, 1946: 87.

#### Psallops niedzwiedzkii

Taxon classificationAnimaliaHemipteraMiridae

Herczek & Popov sp. n.

http://zoobank.org/459FFE5F-85C3-4AD9-9FAB-57282F3F7DC8

##### Material examined.

Holotype: male. X. 65, Forest, Ghana, 2°28'W, 5°25'N, L. R. Cole. D. Leston coll. BM. 1976-509.

##### Diagnosis.

Recognized by the following combination of characters: yellow brown frons and clypeus, a pale reddish-brown, semihyaline corium with a darker embolium, a dark brown cuneus with a lighter apex, metatibiae ferruginous with yellowish apical part. *Psallops niedzwiedzkii* sp. n. is unique due to the ratio (3.78) of its antennae I and II segments, head width to length ratio (2.7), pronotum width to length ratio (2.47), hind tibia to hind femur length ratio (1.12), hind tibia length to pronotum width ratio (1.16) and others. The species is also defined by the distinctive structure of the aedeagus and left paramere (Figs 5, 6, 7).

##### Description.

Male. *Coloration and vestiture*: body generally brownish ferruginous, elongate with brown, semi-erect setae; setae sparsely distributed on pronotum. Dorsal surface weakly smooth, pronotum shagreened. Head, eyes, antennal segments II, rostrum, pronotum, scutellum, cuneus and femora dark brown. Anterior portion of hemelytron and mesoscutum reddish brown. Antennal segment I, frons, clypeus, mandibular and maxillary plates and coxae yellowish brown. Protibiae yellow, mesotibiae brown. Distal 2/3 of metatibiae ferruginous, apical 1/3 yellowish. External tibiae with two rows of brown spines. Membrane slightly smoky, grey.

**Figures 1–4.**
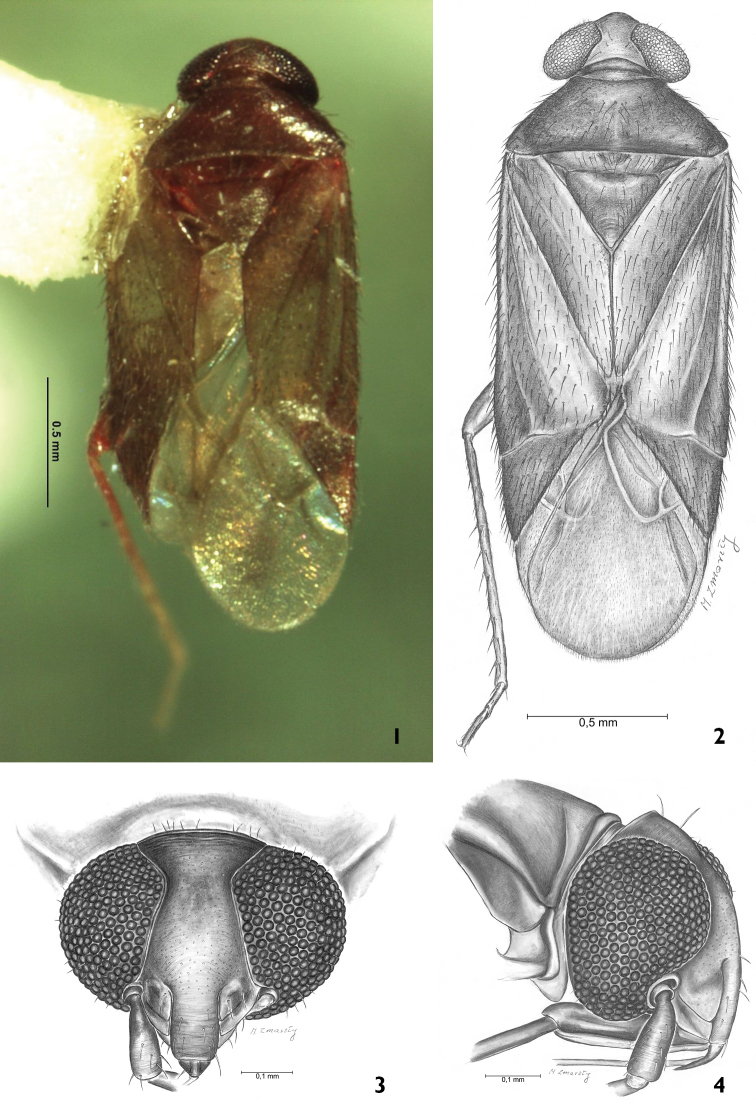
**1, 2** *Psallops niedzwiedzkii* sp. n. dorsal view **3, 4** Front of head, side of head.

**Figures 5–7.**
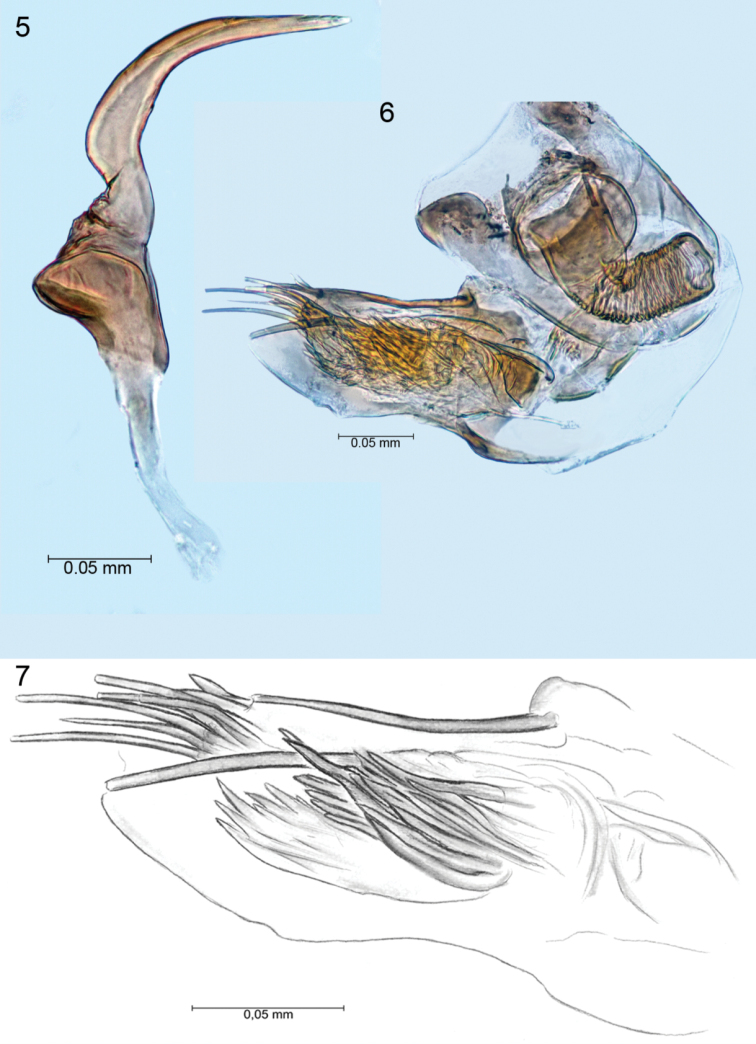
Left paramere, aedeagus, endosoma.

*Structure*: body elongated, 2.67× longer than wide. Head 2.7× wider than long at plane of vertex. Eyes not dissociate at curvatures of vertex (Figs 1, 3). Vertex wide – at narrowest 0.5× as wide as eye. Clypeus relatively short and almost half height of eye, smoothly fused to frons. Mandibular plate wide and short; maxillary plate narrower than buccula. Fovea antennalis situated low, eyes deeply indented. Labium reaches middle coxa. Antennal segments I and II equally thick; segment I 3.78× shorter than II. Pronotum 1.6× longer than head and 2.47× wider than long. Collar clearly visible, but calli weakly marked. Scutellum shorter than pronotum; cumulative length of mesoscutum and scutellum equal to length of claval commissure (1.03). PCu on clavus weakly marked. Corium 3.27× longer than cuneus. Hemelytral membrane with large cell well-developed, with length 2.22× width; smaller cell strongly reduced; vein M forming obvious long process, somewhat shorter than length of large cell (Figs 1, 2). Metafemora, slightly thickened, approximately 3.73× longer than maximum width. Tibiae 2.71× longer than length of tarsus. Tarsi two-segmented, with hind second tarsal segment 2.60× as long as first. Aedeagus delicate and membranous; apical portion of endosoma with eight sublinear strip-like blunt spines with different lengths; medial portion of endosoma with one bunch of sharp tipped spicules (Figs 6, 7). Left paramere scythe-shaped, with sensory lobe with inverted bowl shape; apical process narrow, elongated and subtlety serrate. Paramere body stoutly adjoins apical process (Fig. 5). Right paramere missing.

**Figure 8.**
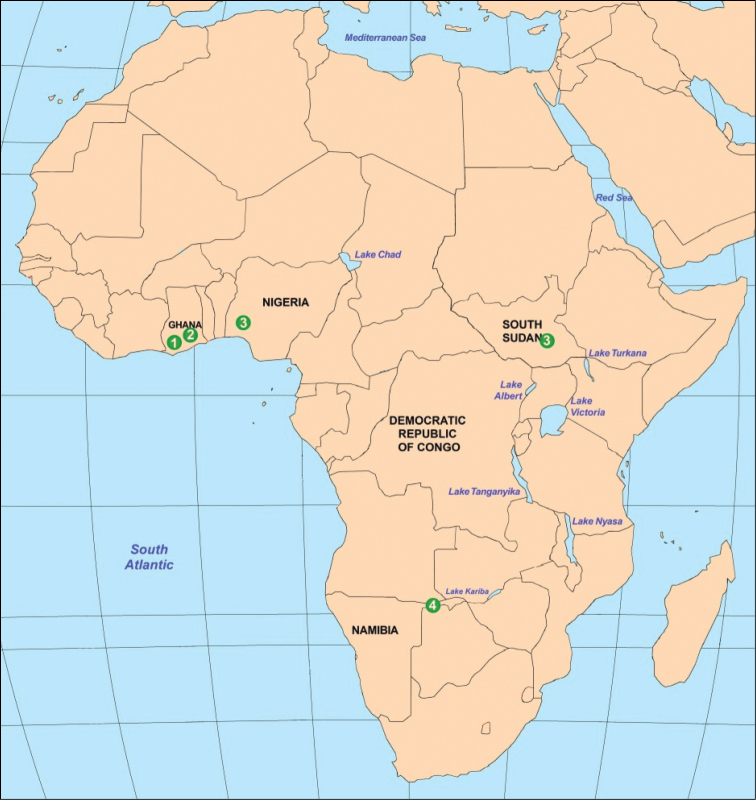
The distribution of African species: **1** *Psallops linnavuorii* **2** *Psallops niedzwiedzkii*, sp. n. **3** *Psallops webbii* **4** *Psallops*, undescribed species

Female. Unknown.

Measurements. male: body length – 2.30; width – 0.86; head: length – 0.20; width – 0.54; height – 0.45; dorsal width of eye – 0.24; width of vertex – 0.12; antennal segments: I – 0.14; II – 0.53; III and IV – missing; rostral segments: I – 0.28; II – 0.26; III – 0.11, IV – 0.13; length of pronotum – 0.32; anterior width – 0.35; posterior width – 0.79; length of mesoscutum – 0.081; length of scutellum – 0.25; length of claval commissurae – 0.42; length of hind femur – 0.82; width – 0.22; hind tibia – 0.92; length of tarsus – 0.34 (I- 0.10; II- 0.26); length of hemelytron – 1,79; length of corium – 1.08; length of cuneus – 0.33.

##### Etymology.

Named in honour of our friend Jacek Niedzwiedzki.

##### Remarks.

*Psallops niedzwiedzkii*, sp. n. is distinguished from other species of *Psallops* primarily by the ratios of the head, antenna, pronotum and legs (see Table 1). The new species also shares certain characters with a few widely distributed species: *Psallops linnavuorii*, *Psallops myiocephalus* Yasunaga, 1999, *Psallops sakarat* Yasunaga, 2010, and *Psallops webbii*. The new species resembles *Psallops sakarat* in the head to vertex width ratio (4.5), head to pronotum width ratio (0.68 and 0.69, respectively) and pronotum to head width ratio (1.46 and 1.44, respectively). The second and third ratios are also typical of *Psallops miocephalus* (the former 0.69 and the latter 1.44). The head width to length ratio and the scutellum to mesoscutum length ratio are similar to those in *Psallops webbii* (2.79 and 3.1, respectively).

The structure of the aedeagus approximates that in *Psallops linnavuorii* although the number of apical spines, shape and arrangement of the bunched medial spicules are different. In *Psallops linnavuorii* the endosoma has two dense bunches of medial sclerotized spicules and four long, blunt tipped spines apically. Moreover, the shape of the left paramere in *Psallops linnavuorii* is similar to *Psallops niedzwiedzkii*; both species are not thickened at the area adjoining the apical process.

### Key to African species

**Table:** 

1	Dorsal part of body brownish to brownish ferruginous, weakly smooth or shagreened. Cuneus brown to dark brown, without yellowish brown distally. Head width to length ratio 2.4–2.7	**2**
–	Dorsal part of body dark brown, slightly crumpled; cuneus dark brown, terminal part yellowish brown. Head width to length ratio 2.79	***Psallops webbii* Herczek & Popov, 2014**
2	Body brownish ferruginous, cuneus dark brown with paler apex. Metafemora dark brown, metatibiae / 3 ferruginous, apical 1/3 yellowish. Head width to length ratio 2.7	***Psallops niedzwiedzkii* sp. n.**
–	Body brownish, cuneus brown, near cuneal fracture whitish and ½ apical part tinged with red. Metafemora brown, metatibiae pale yellow. Head width to length ratio 2.48	***Psallops linnavuorii* Herczek, Popov & Gorczyca, 2016**

#### Psallops webbii

Taxon classificationAnimaliaHemipteraMiridae

Herczek & Popov, 2015

##### Diagnosis.

Male. General colouration of head, pronotum, prosternum, mesosternum, metasternum, first segment of labium, cuneus and anterior margin of hemelytron uniformly dark brown. Mandibular plates, clypeus, antennal segment I, fore and middle legs, tibiae and tarsi of hind legs and labium (except segment I) pale yellow. Eyes reddish-brown with paler edges. Mesoscutum, scutellum and metafemora reddish brown. Corium yellowish brown with anterior portion paler. Corium with small red patches adjoining cuneus. Membrane grey-brown, weakly creased and covered with very fine setae. Body surface slightly crumpled and semi-dull. Mesoscutum and scutellum glossy basally. . Labium long, almost reaching apex of hind coxae; labial segment I reaching middle of mesofemur. Tibiae with sparsely distributed pale spines on external surface; length of spines slightly longer than diameter tibia.

#### Psallops linnavuorii

Taxon classificationAnimaliaHemipteraMiridae

Herczek, Popov & Gorczyca, 2016

##### Diagnosis.

Male. Body generally brownish and elongated. Dorsal surface weakly shagreened with pale, uniformly distributed depressed setae. Head and pronotum dark brown. Frons and clypeus brown. Eyes, antennae and labium infuscate. Mesoscutum, scutellum, clavus, corium, cuneus, coxae and femora brown. Basal part of hemelytron tinged with red, inner apical part and femora pale brown. Cuneus near cuneal fracture whitish and ½ apical part of cuneus tinged with red. Tibiae and tarsi pale yellow. Tibiae with two rows of pale brown spines on external surface; length of spines longer than diameter of tibia.

## Supplementary Material

XML Treatment for Psallops niedzwiedzkii

XML Treatment for Psallops webbii

XML Treatment for Psallops linnavuorii
